# Hierarchical Controlled Hybrid Quantum Communication Based on Six-Qubit Entangled States in IoT

**DOI:** 10.3390/s23229111

**Published:** 2023-11-10

**Authors:** Xiaoyu Hua, Dongfen Li, You Fu, Yonghao Zhu, Yangyang Jiang, Jie Zhou, Xiaolong Yang, Yuqiao Tan

**Affiliations:** College of Computer Science and Cyber Security (Oxford Brookes College), Chengdu University of Technology, Chengdu 610059, China; huaxiaoyu@stu.cdut.edu.cn (X.H.); 2022050889@stu.cdut.edu.cn (Y.F.); 2022050893@stu.cdut.edu.cn (Y.Z.); 2022050894@stu.cdut.edu.cn (Y.J.); 2021020861@stu.cdut.edu.cn (J.Z.); 2021050831@stu.cdut.edu.cn (X.Y.); 2021020897@stu.cdut.edu.cn (Y.T.)

**Keywords:** IoT communication, hierarchical controlled hybrid quantum communication, six-qubit entangled state, quantum secure communication, quantum noise

## Abstract

The rapid development and extensive application of the Internet of Things (IoT) have brought new challenges and opportunities to the field of communication. By integrating quantum secure communication with the IoT, we can provide a higher level of security and privacy protection to counteract security threats in the IoT. In this paper, a hybrid quantum communication scheme using six-qubit entangled states as a channel is proposed for specific IoT application scenarios. This scheme achieves hierarchical control of communication protocols on a single quantum channel. In the proposed scheme, device A transmits data to device B through quantum teleportation, while device B issues control commands to device A through remote quantum state preparation technology. These two tasks are controlled by control nodes C and D, respectively. The transmission of information from device A to device B is a relatively less important task, which can be solely controlled by control node C. On the other hand, issuing control commands from device B to device A is a more crucial task requiring joint control from control nodes C and D. This paper describes the proposed scheme and conducts simulation experiments using IBM’s Qiskit Aer quantum computing simulator. The results demonstrate that the fidelity of the quantum teleportation protocol (QTP) and the remote state preparation protocol (RSP) reach an impressive value of 0.999, fully validating the scheme’s feasibility. Furthermore, the factors affecting the fidelity of the hybrid communication protocol in an IoT environment with specific quantum noise are analyzed. By combining the security of quantum communication with the application scenarios of the IoT, this paper presents a new possibility for IoT communication.

## 1. Introduction

The Internet of Things (IoT) enables communication and data exchange between various devices and systems by connecting them to the Internet. This seamless connectivity allows devices to perform real-time monitoring, remote control, and data sharing, bringing more convenient services and functions to users. However, IoT devices have limited computing power, which poses substantial security challenges for IoT deployment. In the era of quantum communication, these challenges become more severe because some attackers may have quantum computing capabilities, making IoT devices more vulnerable. Unlike traditional secure communication schemes, quantum communication does not rely on computational complexity to guarantee communication security. However, it uses the unique physical properties of quantum mechanics to transmit information, thus achieving secure communication. In recent years, quantum information technology has developed rapidly. In 2022, Xie et al. [1] proposed an asynchronous measurement-device-independent quantum key distribution protocol. In 2023, Zhou et al. [2] implemented an innovative measurement-device-independent quantum key distribution (MDI-QKD) scheme. In 2022, Yin et al. [3] proposed an efficient quantum digital signature (QDS) protocol. In 2023, Liao et al. [4] proposed a continuous-variable quantum secret-sharing scheme based on multi-ring discrete modulation. In 2023, Zhou et al. [5] proposed a hybrid quantum-classical generative adversarial network (HQCGAN). In 2024, Gong et al. [6] designed a quantum convolutional neural network (QCNN) based on pure variational quantum circuits inspired by convolutional neural networks. Quantum communication technology provides new solutions for IoT security issues and application scenarios [7,8,9,10]. In 2021, Maha et al. [11] proposed a novel method that uses quantum key distribution (QKD) technology to encrypt data between IoT devices and servers, which is simple and effective. In the same year, Rajesh Kumar et al. [12] designed a quantum-communication-based IoT security architecture (QIoTSA) and analyzed its advantages and challenges. In 2022, Liu et al. [13] used quantum key distribution (QKD) technology to store quantum keys in IoT devices in advance and used them to encrypt and decrypt IoT-sensitive data.

Quantum teleportation (QTP) and remote state preparation (RSP) are quantum secure communication techniques that use quantum entanglement properties. QTP was first proposed by Bennett and Brassard [14] in 1993 and later experimentally verified by Anton Zeilinger and others in 1997. Since then, QTP has attracted the attention of many researchers and has produced many theoretical and experimental advances and variations in the past thirty years [15,16,17,18]. In 1998, Karlsson and Bourennane [19] pioneered the idea of controlled quantum teleportation (CQTP). In 2008, Chen et al. [20] designed a bidirectional CQTP scheme based on four-qubit entangled states. In 2015, Chen et al. [21] proposed a CQTP scheme based on three-particle partially entangled states. In 2018, Zhou et al. [22] proposed an efficient CQTP scheme based on two-qubit entangled states, which requires the control of a supervisor. In 2020, Li et al. [23] proposed a theoretical CQTP scheme based on seven-qubit entangled states. Remote quantum state preparation (RSP) is an essential branch of quantum communication technology. It differs from quantum teleportation (QTP) in that the sender knows the quantum state to be prepared, while the receiver does not. The sender can perform specific measurements on a certain qubit based on their information, thereby helping the remote receiver recover the original state. In 2000, Lo et al. [24] first proposed the concept of RSP. In recent years, some people have proposed a bidirectional hybrid controlled quantum communication (BHCQC) scheme that combines QTP and RSP. In 2017, Fang et al. [25] used a five-qubit Brown state as the channel to give a deterministic BHCQC protocol for any single-qubit state. In the same year, Sang et al. [26] also used a five-qubit cluster state as the channel to realize a deterministic BHCQC protocol for any single-qubit state. In 2018, Ma et al. [27] used a six-qubit entangled state to perform BHCQC. Hierarchical quantum communication is a novel means of multiparty quantum communication in recent years. It uses different levels of quantum entangled states to achieve quantum information transmission among multiple parties. In 2010, Wang et al. [28] proposed the first scheme of multiparty hierarchical controlled quantum teleportation (HCQTP) using four-particle entangled states. In 2013, Shukla and Pathak et al. [29] performed similar work using four-particle entangled states. In 2017, Shukla et al. [30] proposed a protocol for the hierarchical joint remote preparation of any single-qubit state using five-particle cluster states. In 2018, Chen et al. [31] realized a deterministic hierarchical remote preparation of any single-qubit state using six-particle partially entangled states. In 2020, Wang et al. [32] used four-particle states as the channel to design a single-qubit state hierarchical CQTP scheme. In 2021, Ma and Wang [33] used two five-particle cluster states to carry out a hierarchical controlled remote preparation of two-qubit states.

In the previous BHCQC schemes, quantum teleportation and remote quantum state preparation were regarded as equally important, lacking control flexibility, and making them unsuitable for specific IoT scenarios. In order to migrate BHCQC to the IoT, this study attempts to introduce the concept of hierarchical control into the hybrid communication protocol, assigning different levels of importance to quantum teleportation and remote quantum state preparation. Thus, this paper proposes a hierarchical controlled hybrid quantum communication (HCHQC) scheme utilizing a six-qubit entangled state as the quantum channel. Alice wishes to transmit an unknown quantum state to Bob, while simultaneously, Bob desires to prepare a known quantum state on Alice’s side remotely. This process is controlled by supervisors Charlie and David. The state Alice wants to transmit to Bob is a less critical task and can be solely controlled by supervisor Charlie. However, the state that Bob intends to prepare for Alice remotely is a more important task and must be jointly controlled by supervisors Charlie and David.

The remaining structure of this paper is as follows: Section 2 presents the application scenarios of the HCHQC protocol in the IoT and provides a detailed description of the HCHQC protocol. In Section 3, we employ IBM’s Qiskit Aer quantum computing simulator to conduct simulation experiments to validate the proposed scheme’s feasibility. In Section 4, we introduce the presence of amplitude-damping noise and phase-damping noise environments and analyze the factors that impact the fidelity of the HCHQC protocol in specific noise environments. Finally, Section 5 provides a comprehensive summary of this paper.

## 2. Hierarchical Controlled Hybrid Quantum Communication Scheme in the IoT

### 2.1. Application Scenario

The Internet of Things (IoT) is a framework that uses internet technology to connect various smart devices and achieve information sharing and interaction. The IoT is widely used in many fields, such as smart homes, intelligent transportation, smart medical care, etc. However, the IoT also faces security issues, such as device identity authentication, data confidentiality and integrity, network defence, etc. To cope with these challenges, the quantum secret communication protocol provides a new solution. The quantum secret communication protocol is a technology that uses quantum mechanics principles, such as quantum superposition, quantum entanglement, quantum no-cloning, etc., to achieve information-secure transmission and encryption. It can effectively resist eavesdropping and interference from third parties and ensure the security and reliability of IoT communication. This paper will discuss a scenario that uses a quantum secret communication protocol to protect IoT communication security.

**Figure 1:**
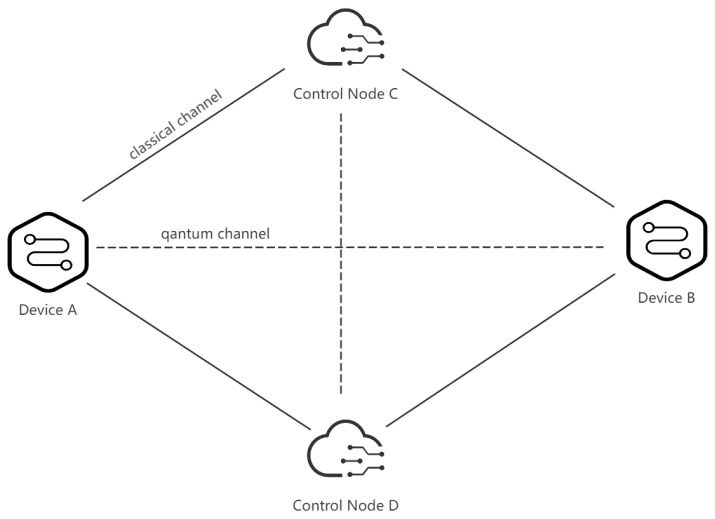
Information exchange and control between different devices in the IoT system through classical and quantum communication channels.

Device A and device B are edge devices in the IoT that are close to the data source or the user and can process and analyze data locally. Device A is equipped with multiple sensors, which can collect environmental data, such as temperature, pressure, etc. Device B can receive the environmental status of other edge devices and issue corresponding instructions according to the status change. Control node C and control node D are cloud computing devices that are responsible for the authentication and control services of the IoT and ensure the security of the IoT. These devices transmit classical data through classical network channels and exchange quantum information through quantum channels, as shown in Figure 1.

Device A must securely transmit the environmental state data (such as temperature, pressure, etc.) to another device, device B, within the IoT system. This transmission of the environmental state exists at a general level of security within the entire IoT system; thus necessitating authorization solely from control node C. Device A can encode the intended state information into a quantum state, denoted as state *a*, and employ controlled quantum teleportation to transmit this quantum state to device B. By measuring the received quantum state *a*, device B can retrieve the environmental state information from device A’s side. Conversely, device B controls device A, allowing it to make determinations based on the transmitted environmental state data and issue control commands to device A. Considering the security of the IoT system, this manipulation operates at a higher level of protection throughout the entire IoT system, requiring joint authorization from control nodes C and D. Device B can encode the desired instructions into a quantum state, denoted as state *b*, and utilizing remote quantum state preparation under the shared control of control nodes C and D, create a quantum state on device A’s side that matches the state of quantum state *b*. Device A can measure the quantum state to decode the instruction.

Based on the scenario of using a quantum secret communication protocol to ensure the security of IoT communication, this paper proposes an HCHQC protocol that uses a six-qubit entangled state as the quantum channel and realizes the layered control of two different communication protocols, quantum invisible transmission, and remote quantum state preparation, on one quantum channel. In the communication scheme, device A and device B represent Alice and Bob, respectively, and control node C and control node D represent Charlie and David, respectively. The following Section 2, will detail this hybrid quantum communication scheme that uses a six-qubit entangled state as the quantum channel for the IoT.

### 2.2. Specific Communication Plan

The communication scheme introduced in this section has the following advantages: (1) flexible communication, which can be controlled according to the task requirements and security levels; (2) quantum resource saving, which can complete two tasks with one six-qubit entangled state; (3) high communication efficiency, which can transmit and reconstruct states with a small amount of classical information and quantum operations. Next, we will explain this scheme in detail.

Assume Alice has an arbitrary unknown single-qubit state, denoted as:

(1) $${|\varphi \rangle }_{a}=\alpha_{1}|0\rangle +\alpha_{2}|1\rangle$$

where $\alpha_{1}$ and $\alpha_{2}$ are complex numbers satisfying ${\left|\alpha_{1}\right|}^{2}+{\left|\alpha_{2}\right|}^{2}=1$.

Alice wants to teleport an unknown single qubit state |φ〉 to Bob using quantum teleportation. Meanwhile, Bob wants to prepare a known single qubit state |ϕ〉 on Alice’s side through remote state preparation. The state of |ϕ〉 can be written as:

(2) $${|\phi \rangle }_{b}=\beta_{1}|0\rangle +\beta_{2}|1\rangle$$

where $\beta_{1}$ and $\beta_{2}$ are real numbers satisfying ${\left|\beta_{1}\right|}^{2}+{\left|\beta_{2}\right|}^{2}=1$.

At this point, assuming that the quantum channel shared by Alice, Bob, Charlie, and David is a six-qubit entangled quantum state, it can be expressed as:

(3) $${|\psi \rangle }_{A_{1}B_{1}A_{2}B_{2}CD}=\frac{1}{2}{\left(|000000\rangle -|001111\rangle +|110010\rangle -|111101\rangle \right)}_{A_{1}B_{1}A_{2}B_{2}CD}$$

Among them, qubits $A_{1}$ and $A_{2}$ belong to Alice, qubits $B_{1}$ and $B_{2}$ belong to Bob, qubit *C* belongs to Charlie, and qubit *D* belongs to David. The quantum state of the whole system can be expressed as follows:

(4) $$\begin{array}{cc} {|\tau \rangle }_{aA_{1}B_{1}A_{2}B_{2}CD}= & {|\varphi \rangle }_{a}⊗{|\psi \rangle }_{A_{1}B_{1}A_{2}B_{2}CD}\\ = & \frac{1}{2}{\left(\alpha_{1}|0\rangle +\alpha_{2}|1\rangle \right)}_{a}\\ \; & ⊗{\left(|000000\rangle -|001111\rangle +|110010\rangle -|111101\rangle \right)}_{A_{1}B_{1}A_{2}B_{2}CD} \end{array}$$

where ⊗ represents the tensor product. We now introduce the hybrid quantum communication scheme in four steps.

Step 1:

Alice performs a joint measurement on two qubits *a* and $A_{1}$ she possesses, using the Bell measurement basis given by the following expression:

(5) $$\begin{array}{cc} {|\Psi^{1}\rangle }_{a,A_{1}} & =\frac{1}{\sqrt{2}}\left(|00\rangle +|11\rangle \right)\\ {|\Psi^{2}\rangle }_{a,A_{1}} & =\frac{1}{\sqrt{2}}\left(|00\rangle -|11\rangle \right)\\ {|\Psi^{3}\rangle }_{a,A_{1}} & =\frac{1}{\sqrt{2}}\left(|01\rangle +|10\rangle \right)\\ {|\Psi^{4}\rangle }_{a,A_{1}} & =\frac{1}{\sqrt{2}}\left(|01\rangle -|10\rangle \right) \end{array}$$

Alice can obtain one of the four possible measurement results with equal probability, and the remaining qubits $B_{1}$, $A_{2}$, $B_{2}$, *C*, and *D* will collapse into one of the corresponding four states $\langle \Psi^{1}||\tau \rangle$, $\langle \Psi^{2}||\tau \rangle$, $\langle \Psi^{3}||\tau \rangle$, or $\langle \Psi^{4}||\tau \rangle$, as shown in Table 1.

Step 2:

At the same time, Bob performs projection measurement on his qubit $B_{2}$, and the measurement basis is:

(6) $$\begin{array}{cc} {|\Phi^{1}\rangle }_{B_{2}} & =\beta_{1}|0\rangle +\beta_{2}|1\rangle \\ {|\Phi^{2}\rangle }_{B_{2}} & =\beta_{2}|0\rangle -\beta_{1}|1\rangle \end{array}$$

Since the coefficients $\beta_{1}$ and $\beta_{2}$ are known to Bob, such a set of measurement bases can be obtained. The projection measurements and the states of the remaining qubits are shown in Table 2 below.

Step 3:

Alice needs Charlie’s help to complete the task of quantum teleporting the quantum state |φ〉 to Bob using quantum entanglement. Suppose Charlie agrees to assist Alice and Bob. In that case, he needs to perform a von Neumann measurement on his qubit *C* on the basis $\left\{|\nu^{1}\rangle ,|\nu^{2}\rangle \right\}$ and communicate the measurement result to Bob through the classical communication channel. The measurement basis $\left\{|\nu^{1}\rangle ,|\nu^{2}\rangle \right\}$ is as follows:

(7) $$\begin{array}{cc} |\nu^{1}\rangle & =\frac{1}{\sqrt{2}}\left(|0\rangle +|1\rangle \right)\\ |\nu^{2}\rangle & =\frac{1}{\sqrt{2}}\left(|0\rangle -|1\rangle \right) \end{array}$$

Now suppose a situation exists where, before Charlie performs a single-qubit von Neumann measurement, the measurement results of Alice and Bob are $|\Psi^{1}\rangle$ and $|\Phi^{1}\rangle$, respectively, then the remaining qubits will collapse to the state ${\left(\alpha_{1}\beta_{1}|0000\rangle -\alpha_{1}\beta_{2}|0111\rangle +\alpha_{2}\beta_{1}|1010\rangle -\alpha_{2}\beta_{2}|1101\rangle \right)}_{B_{1}A_{2}CD}$. After Charlie performs the von Neumann measurement on qubit C, if the obtained measurement result is $|\nu^{2}\rangle$, the state of the remaining qubits will collapse to:

(8) $$\langle \nu^{2}|\langle \Phi^{1}|\langle \Psi^{1}||\tau \rangle ={\left(\alpha_{1}\beta_{1}|000\rangle +\alpha_{1}\beta_{2}|011\rangle -\alpha_{2}\beta_{1}|100\rangle -\alpha_{2}\beta_{2}|111\rangle \right)}_{B_{1}A_{2}D}$$

We can go one step further and write the equation as follows:

(9) $$\langle \nu^{2}|\langle \Phi^{1}|\langle \Psi^{1}||\tau \rangle ={\left(\alpha_{1}|0\rangle -\alpha_{2}|1\rangle \right)}_{B_{1}}⊗{\left(\beta_{1}|00\rangle +\beta_{2}|11\rangle \right)}_{A_{2}D}$$

It can be clearly seen that after Bob learns the measurement result of Charlie through the classical channel, he only needs to perform the $\sigma_{z}$ gate operation on the qubit $B_{1}$ to correct the quantum state. Then he can obtain the quantum state |φ〉. However, the qubits $A_{2}$ and *D* are still entangled, so Alice cannot obtain the state |ϕ〉 prepared remotely by Bob. The other cases and the unitary operations corresponding to each case are shown in Table 3.

Step 4:

To remotely prepare the quantum state |ϕ〉 from Bob to Alice, supervisors Charlie and David are needed. David must perform the qubit *D* single-qubit von Neumann measurements. Once Alice receives the measurement results from Charlie and David through the classical channel, she can perform the corresponding unitary operation on qubit $A_{2}$ to obtain the desired quantum state |ϕ〉 that Bob intends to prepare.

In Step 3’s example, if David’s measurement result is $|\nu^{1}\rangle$, then Alice will obtain the collapsed state of qubit $A_{2}$ as $\beta_{1}|0\rangle +\beta_{2}|1\rangle$. To reconstruct the quantum state |ϕ〉, Alice can perform the unitary operation *I* on $A_{2}$. On the other hand, if David’s measurement result is $|\nu^{2}\rangle$, then Alice will observe the collapsed state of qubit $A_{2}$ as $\beta_{1}|0\rangle -\beta_{2}|1\rangle$. To reconstruct the quantum state |ϕ〉, Alice can perform the unitary operation $\sigma_{z}$ on $A_{2}$. The details of David’s measurement results, the state of qubit $A_{2}$, and Alice’s unitary operation are presented in Table 4.

The four unitary operations used in the transmission process are:

(10) $$\begin{array}{c} I=|0\rangle \langle 0|+|1\rangle \langle 1|\\ \sigma_{z}=|0\rangle \langle 0|-|1\rangle \langle 1|\\ \sigma_{x}=|0\rangle \langle 1|+|1\rangle \langle 0|\\ i\sigma_{y}=|0\rangle \langle 1|-|1\rangle \langle 0| \end{array}$$

The above steps illustrate how a hybrid quantum communication system is implemented using a six-qubit entangled state as a channel. During the transmission process, Alice sends the state of qubit *a* to Bob via quantum teleportation, which Charlie supervises. Simultaneously, Bob prepares a qubit $A_{2}$ in the same state as qubit *b* on Alice’s side through remote state preparation, which Charlie and David jointly supervise. This approach allows different communication protocols to be controlled on a quantum channel, improving communication flexibility.

## 3. Experimental Verification

To test the feasibility of our proposed hybrid quantum communication scheme in IoT communication, we conducted a simulation experiment using IBM’s Qiskit Aer (0.13.0) quantum computing simulator. Qiskit Aer is an open-source quantum computing software package that can simulate quantum circuits on different noise models and backend devices. We used the statevector-simulator in Qiskit Aer as the backend device, which can give the final state vector after the quantum circuit runs. We built a quantum circuit with seven qubits and sixteen classical bits, completed the four steps in the scheme, and recorded the measurement results and correction operations of Alice, Bob, Charlie, and David. Next, we will show the whole process and results of the experiment.

**Figure 2:**
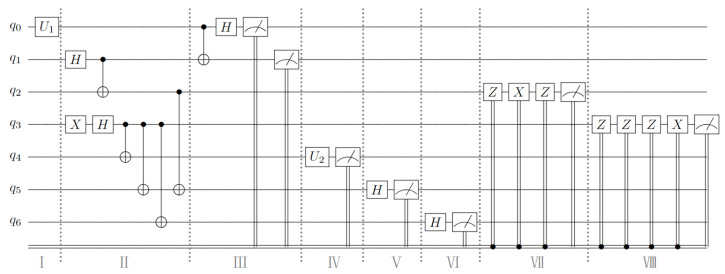
Using the Qiskit Aer quantum computing simulator to implement quantum circuit design for HCHQC protocols.

The complete process of the experiment is shown in Figure 2, where $q_{1}$∼$q_{6}$ is a six-qubit quantum channel, and $q_{0}$ is a single-qubit quantum state; their initial states are all |0〉. Part I shows the quantum circuit that generates arbitrary single-qubit states through $U_{1}$ gates. In this experiment, we set the parameters of the $U_{1}$ gate to (θ=π/2, φ=0, λ=π). We can obtain a quantum state of ${|\varphi \rangle }_{a}=\frac{1}{\sqrt{2}}\left(|0\rangle +|1\rangle \right)$ through the unitary operation of the $U_{1}$ gate. This qubit is utilized for experimental verification purposes. Part II is the process of preparing quantum channels, showing the use of *H* gates, *X* gates, and CNOT gates to generate six-qubit entangled states. Part III shows Alice’s process of performing Bell state measurements on qubits *a* and $A_{1}$. In this process, according to different measurement results, Alice will obtain two classical bit information. The measurement results and corresponding classical bit encoding are shown in Table 5 below. The same goes for Bob, Charlie, and David.

Part IV is the circuit where Bob performs projection measurement on his qubit $B_{2}$. In this experiment, we set the quantum state that Bob wants to transmit as ${|\phi \rangle }_{b}=\frac{1}{\sqrt{3}}|0\rangle +\frac{2}{\sqrt{3}}|1\rangle )$. Therefore, the quantum gate $U_{2}$ used for projection measurement can be represented by the following matrix:

$$U_{2}=\left(\begin{array}{cc} \frac{1}{\sqrt{3}} & \frac{2}{\sqrt{3}}\\ \frac{2}{\sqrt{3}} & -\frac{1}{\sqrt{3}} \end{array}\right)$$

Part V and Part VI show the circuits used by Charlie and David to perform the von Neumann measurements. Parts VII and VIII represent the process of reconstructing quantum information by Bob and Alice according to the classical bit encoding by the corresponding unitary operations.

**Figure 3:**
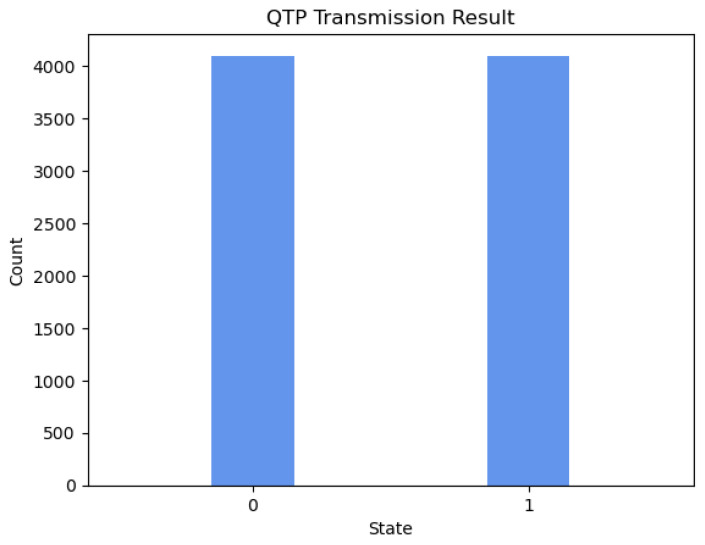
Probability distribution of the results of 8192 experiments of HCHQC protocol on Qiskit Aer when Alice performs QTP to Bob.

**Figure 4:**
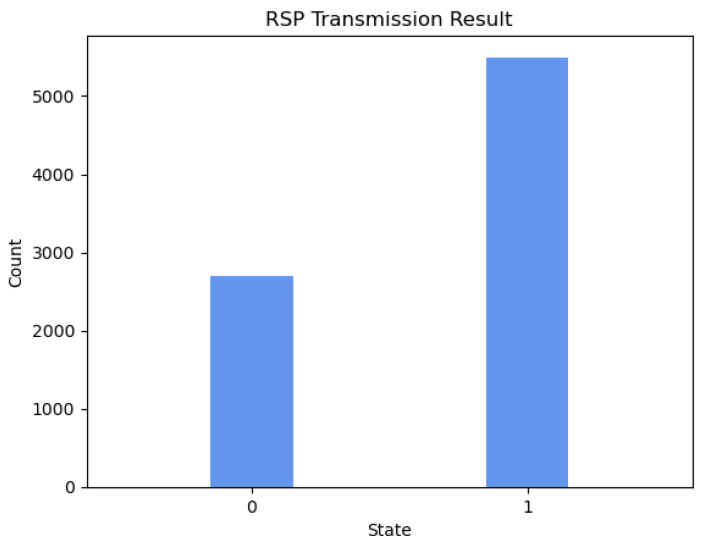
Probability distribution of the results of 8192 experiments of HCHQC protocol on Qiskit Aer when Bob performs RSP to Alice.

Qiskit Aer is a high-performance quantum computing simulator with realistic noise models. Using several different simulation methods, it provides an interface to run quantum circuits with or without noise. By running the above quantum circuit in the Qiskit Aer simulator, the probability distribution of the qubit states obtained by Alice and Bob can be obtained. The following Figure 3 and Figure 4 shows the experimental results of 8192 experiments on Qiskit Aer.

According to Table 6, the qubit state obtained by Alice and Bob can be written as Equation (11), where ${|\varphi \rangle }_{B_{1}}$ represents the QTP simulated state received by Bob calculated based on the actual transmission result, and ${|\phi \rangle }_{A_{2}}$ represents the RSP simulated state received by Alice calculated based on the actual transmission result.

(11) $$\begin{array}{cc} {|\varphi \rangle }_{B_{1}} & =\sqrt{0.5002}|0\rangle +\sqrt{0.4997}|1\rangle \\ {|\phi \rangle }_{A_{2}} & =\sqrt{0.3291}|0\rangle +\sqrt{0.6708}|1\rangle \end{array}$$

Fidelity is a metric that assesses the efficacy of quantum communication protocols. It signifies the degree of resemblance between the transmitted quantum state and the original quantum state. Generally speaking, higher fidelity indicates a more successful quantum communication protocol. In this scheme, we can calculate the fidelity of QTP from Alice to Bob and RSP from Bob to Alice. The calculation formulas are (12) and (13), respectively:

(12) $$\begin{array}{cc} F_{QTP}={\langle \varphi |}_{a}\rho_{1}{|\varphi \rangle }_{a}= & \left(\frac{\sqrt{2}}{2}\langle 0|+\frac{\sqrt{2}}{2}\langle 1|\right)\left(\sqrt{0.5002}|0\rangle +\sqrt{0.4997}|1\rangle \right)\\ & \left(\sqrt{0.5002}\langle 0|+\sqrt{0.4997}\langle 1|\right)\left(\frac{\sqrt{2}}{2}|0\rangle +\frac{\sqrt{2}}{2}|1\rangle \right)\approx 0.999 \end{array}$$

(13) $$\begin{array}{cc} F_{RSP}={\langle \phi |}_{b}\rho_{2}{|\phi \rangle }_{b}= & \left(\frac{1}{\sqrt{3}}\langle 0|+\frac{2}{\sqrt{3}}\langle 1|\right)\left(\sqrt{0.3291}|0\rangle +\sqrt{0.6708}|1\rangle \right)\\ & \left(\sqrt{0.3291}\langle 0|+\sqrt{0.6708}\langle 1|\right)\left(\frac{1}{\sqrt{3}}|0\rangle +\frac{2}{\sqrt{3}}|1\rangle \right)\approx 0.999 \end{array}$$

where $F_{QTP}$ represents the fidelity of quantum teleportation from Alice to Bob, $F_{RSP}$ represents the fidelity of remote state preparation from Bob to Alice, $\rho_{1}={|\varphi \rangle }_{B_{1}}\langle \varphi |$ represents the density operator of the QTP simulated state, and $\rho_{2}={|\phi \rangle }_{A_{2}}\langle \phi |$ represents the density operator of the RSP simulated state. According to the calculation results of the above formulas, we can see that, in our designed experiment, the fidelity of the quantum state transmitted by Alice to Bob via quantum teleportation (QTP) is as high as 0.999, close to the ideal value of 1, which demonstrates the validity of QTP in the hybrid quantum communication protocol; similarly, the fidelity of the quantum state prepared by Bob for Alice via remote quantum state preparation (RSP) is also 0.999, close to the ideal value of 1, which demonstrates the validity of RSP in the hybrid quantum communication protocol.

## 4. Impact of Quantum Noise on Hybrid Quantum Communication Schemes

Quantum secret communication uses the characteristics of quantum mechanics to achieve high-security information transmission. However, there are also some challenges in applying quantum secret communication in the IoT, such as the interference of quantum noise. Quantum noise refers to the environmental factors or device defects in the quantum channel, which cause the transmitted quantum state to change or be in error. This affects the efficiency and security of quantum secret communication and may even cause communication interruption or cracking. There are several main types of quantum noise in the IoT:

1. Amplitude-damping noise: This particular noise engenders the disappearance or attenuation of photons, thereby diminishing both the signal strength and the signal-to-noise ratio. Amplitude-damping noise primarily arises from factors such as fibre-optic transmission losses, reflection in optical devices, and scattering;
2. Phase-damping noise: This particular noise induces random variations in the phase of photons, thereby disrupting the quantum superposition and entanglement states. Phase-damping noise primarily stems from factors such as temperature fluctuations, mechanical vibrations, and electromagnetic interference;
3. Displacement noise: This noise engenders random displacements in the position of photons, thereby altering their wavelength or frequency. Displacement noise primarily arises from factors such as the Doppler effect, fibre dispersion, and nonlinear effects;
4. Rotational noise: This particular noise induces random rotations in the polarization direction of photons, thereby altering their polarization state. Rotational noise primarily stems from factors such as fibre birefringence, the Faraday effect, and magnetic fields.

In this section, we have chosen two exemplary forms of quantum noise: amplitude-damping noise and phase-damping noise. We shall discuss the fidelity of communication in the IoT communication scenario for the HCHQC protocol in the presence of these two types of quantum noise. The Kraus operator for amplitude-damping noise is expressed as:

(14) $$E_{0}^{A}=\left[\begin{array}{cc} 1 & 0\\ 0 & \sqrt{1-\eta_{A}} \end{array}\right],\;E_{1}^{A}=\left[\begin{array}{cc} 0 & \sqrt{\eta_{A}}\\ 0 & 0 \end{array}\right]$$

where $\eta_{A}$ is the error probability caused by the amplitude-damping noise of the qubit. The Kraus operator for the phase-damping noise is expressed as:

(15) $$E_{0}^{P}=\sqrt{1-\eta_{P}}I,\;E_{1}^{P}=\sqrt{\eta_{P}}\left[\begin{array}{cc} 1 & 0\\ 0 & 0 \end{array}\right],\;E_{2}^{P}=\sqrt{\eta_{P}}\left[\begin{array}{cc} 0 & 0\\ 0 & 1 \end{array}\right]$$

Here, $\eta_{P}$ represents the strength of the noise, and *I* is the identity matrix. In the presence of quantum noise, the input of a pure state will be converted into a mixed state, which can be expressed more conveniently in the form of a density matrix. Therefore, the channel in the pure state can be written as $\rho ={|\psi \rangle }_{A_{1}B_{1}A_{2}B_{2}CD}\langle \psi |$ such a form of the density matrix.

Assume that the channel particles are prepared by the supervisor David, who then sends $A_{1}$, $A_{2}$ to Alice, $B_{1}$, $B_{2}$ to Bob, and *C* to Charlie. To simplify the analysis model, assume that the particle *C* sent by David to Charlie is not affected by noise, the two particles received by Alice are affected by the same noise, and the two particles received by Bob are affected by the same noise. The following formula can describe the influence of noise on the channel:

(16) $$\begin{array}{cc} \xi^{r}\left(\rho \right)= & \sum_{m,n}\left(E_{m}^{r,A_{1}}\right)\left(E_{n}^{r,B_{1}}\right)\left(E_{m}^{r,A_{2}}\right)\left(E_{n}^{r,B_{2}}\right)\\ \; & \rho {\left(E_{m}^{r,A_{1}}\right)}^{†}{\left(E_{n}^{r,B_{1}}\right)}^{†}{\left(E_{m}^{r,A_{2}}\right)}^{†}{\left(E_{n}^{r,B_{2}}\right)}^{†} \end{array}$$

where r∈{A,P}. When r=A, the formula describes the amplitude-damping noise; when r=P, the formula describes the phase-damping noise. The subscript m,n∈{0,1,2} of *E* indicates the Kraus operators corresponding to each noise. The second superscript of *E* indicates which qubit of the channel the corresponding noise operator acts on.

When the quantum channel is affected by these two different noises, it will become the corresponding mixed state:

(17) $$\begin{array}{cc} \xi^{A}\left(\rho \right)= & \frac{1}{4}\{[|000000\rangle -\left(1-\eta_{A}\right)|001111\rangle +\\ \; & \left(1-\eta_{A}\right)|110010\rangle -{\left(1-\eta_{A}\right)}^{2}|111101\rangle ]\times \\ \; & [\langle 000000|-\left(1-\eta_{A}\right)\langle 001111|+\\ \; & \left(1-\eta_{A}\right)\langle 110010|-{\left(1-\eta_{A}\right)}^{2}\langle 111101|]+\\ \; & \left[-\eta_{A}\left(1-\eta_{A}\right)|101001\rangle \right]\times \left[-\eta_{A}\left(1-\eta_{A}\right)\langle 101001|\right]+\\ \; & \left[-\eta_{A}\left(1-\eta_{A}\right)|010101\rangle \right]\times \left[-\eta_{A}\left(1-\eta_{A}\right)\langle 010101|\right]+\\ \; & \left[-\eta_{A}^{2}|000001\rangle \right]\times \left[-\eta_{A}^{2}\langle 000001|\right]\} \end{array}$$

(18) $$\begin{array}{cc} \xi^{P}\left(\rho \right)= & \frac{1}{4}\{[{\left(1-\eta_{P}\right)}^{2}|000000\rangle -{\left(1-\eta_{P}\right)}^{2}|001111\rangle +\\ \; & {\left(1-\eta_{P}\right)}^{2}|110010\rangle ]-{\left(1-\eta_{P}\right)}^{2}|111101\rangle ]\times \\ \; & [{\left(1-\eta_{P}\right)}^{2}\langle 000000|-{\left(1-\eta_{P}\right)}^{2}\langle 001111|+\\ \; & {\left(1-\eta_{P}\right)}^{2}\langle 110010|]-{\left(1-\eta_{P}\right)}^{2}\langle 111101|]+\\ \; & \eta_{P}^{2}\left(2-\eta_{P}^{2}\right)|000000\rangle \langle 000000|+\\ \; & \eta_{P}^{2}\left(2-\eta_{P}^{2}\right)|111101\rangle \langle 111101|\} \end{array}$$

When Alice, Bob, Charlie, and David complete the communication operation, the final state density matrix can be expressed as follows:

(19) $$\rho_{\mathrm{out}\;}^{r}={\mathrm{Tr}}_{aA_{1}B_{2}CD}\left\{U_{ijkl}\left[\rho_{a}⊗\xi^{r}\left(\rho \right)\right]U_{ijkl}^{†}\right\}$$

In the above formula, $\rho_{a}=\left(\alpha_{1}|0\rangle +\alpha_{2}|1\rangle \right)\left(\alpha_{1}\langle 0|+\alpha_{2}\langle 1|\right)$, $U_{ijkl}$ is:

(20) $$\begin{array}{cc} U_{ijkl}= & \left\{I_{a}⊗I_{A_{1}}⊗\sigma_{B_{1}}^{ijk}⊗\sigma_{A_{2}}^{ijkl}⊗I_{B_{2}}⊗I_{C}⊗I_{D}\right\}\\ \; & \left\{I_{a}⊗I_{A_{1}}⊗I_{B_{1}}⊗I_{A_{2}}⊗I_{B_{2}}⊗I_{C}⊗{\langle \nu^{l}|}_{D}\right\}\\ \; & \left\{I_{a}⊗I_{A_{1}}⊗I_{B_{1}}⊗I_{A_{2}}⊗I_{B_{2}}⊗{\langle \nu^{k}|}_{C}⊗I_{D}\right\}\\ \; & \left\{I_{a}⊗I_{A_{1}}⊗I_{B_{1}}⊗I_{A_{2}}⊗{\langle \Phi^{j}|}_{B_{2}}⊗I_{C}⊗I_{D}\right\}\\ \; & \left\{{\langle \Psi^{i}|}_{aA_{1}}⊗I_{B_{1}}⊗I_{A_{2}}⊗I_{B_{2}}⊗I_{C}⊗I_{D}\right\} \end{array}$$

Here, i∈{1,2,3,4}, ${\langle \Psi^{i}|}_{aA_{1}}$ represents the result of the Bell state measurement on qubit $a,A_{1}$; j∈{1,2}, ${\langle \Phi^{j}|}_{B_{2}}$ represents the result obtained by custom base projection measurement on qubit $B_{2}$; k,l∈{1,2}, $\langle \nu^{k}|,\langle \nu^{l}|$ represent the results obtained by von Neumann measurement of qubits *C* and *D*, respectively; while $\sigma_{B_{1}}^{ijk},\sigma_{A_{2}}^{ijkl}$ represent the corresponding unitary operations on qubits $B_{1},A_{2}$ according to the measurement results. Through the calculation of the above formula, the density matrix of the resulting state obtained by executing the HCHQC protocol under the influence of two noise environments can be obtained:

(21) $$\;\begin{array}{cc} \rho_{\mathrm{out}\;}^{A}= & \frac{1}{32}\{[\alpha_{1}\left(\beta_{1}|00\rangle -\beta_{2}\left(1-\eta_{A}\right)|01\rangle \right)+\\ & \alpha_{2}\left(\beta_{1}\left(1-\eta_{A}\right)|10\rangle -\beta_{2}{\left(1-\eta_{A}\right)}^{2}|11\rangle \right)]\times \\ \; & [\alpha_{1}\left(\beta_{1}\langle 00|-\beta_{2}\left(1-\eta_{A}\right)\langle 01|\right)+\\ & \alpha_{2}\left(\beta_{1}\left(1-\eta_{A}\right)\langle 10|-\beta_{2}{\left(1-\eta_{A}\right)}^{2}\langle 11|\right)]+\\ \; & \alpha_{2}^{2}\beta_{1}^{2}\eta_{A}^{2}{\left(1-\eta_{A}\right)}^{2}|01\rangle \langle 01|+\\ \; & \alpha_{1}^{2}\beta_{2}^{2}\eta_{A}^{2}{\left(1-\eta_{A}\right)}^{2}|10\rangle \langle 10|+\\ \; & \alpha_{1}^{2}\beta_{1}^{2}\eta_{A}^{4}|00\rangle \langle 00|\} \end{array}$$

(22) $$\begin{array}{cc} \rho_{\mathrm{out}\;}^{A}= & \frac{1}{32}\{[\alpha_{1}\left(\beta_{1}{\left(1-\eta_{P}\right)}^{2}|00\rangle -\beta_{2}{\left(1-\eta_{P}\right)}^{2}|01\rangle \right)+\\ & \alpha_{2}\left(\beta_{1}{\left(1-\eta_{P}\right)}^{2}|10\rangle -\beta_{2}{\left(1-\eta_{P}\right)}^{2}|11\rangle \right)]\times \\ \; & [\alpha_{1}\left(\beta_{1}{\left(1-\eta_{P}\right)}^{2}\langle 00|-\beta_{2}{\left(1-\eta_{P}\right)}^{2}\langle 01|\right)+\\ \; & \alpha_{2}\left(\beta_{1}{\left(1-\eta_{P}\right)}^{2}\langle 10|-\beta_{2}{\left(1-\eta_{P}\right)}^{2}\langle 11|\right)]+\\ \; & \alpha_{1}^{2}\beta_{1}^{2}\eta_{P}^{2}\left(2-\eta_{P}^{2}\right)|00\rangle \langle 00|+\\ \; & \alpha_{2}^{2}\beta_{2}^{2}\eta_{P}^{2}\left(2-\eta_{P}^{2}\right)|11\rangle \langle 11|\} \end{array}$$

With the density matrix of the communication results in the above two noise environments, this can be brought into the formula:

(23) $$F_{r}={\langle \varphi |}_{B_{1}}{\langle \phi |}_{A_{2}}\rho_{\mathrm{out}\;}^{r}{|\varphi \rangle }_{B_{1}}{|\phi \rangle }_{A_{2}}$$

The corresponding communication fidelity can be obtained:

(24) $$\begin{array}{cc} F_{A}= & \frac{1}{32}\{{\left[\alpha_{1}^{2}\beta_{1}^{2}+\alpha_{1}^{2}\beta_{2}^{2}\left(1-\eta_{A}\right)+\alpha_{2}^{2}\beta_{1}^{2}\left(1-\eta_{A}\right)+\alpha_{2}^{2}\beta_{2}^{2}{\left(1-\eta_{A}\right)}^{2}\right]}^{2}\\ & +2\alpha_{1}^{2}\beta_{1}^{2}\alpha_{2}^{2}\beta_{2}^{2}\eta_{A}^{2}{\left(1-\eta_{A}\right)}^{2}+\alpha_{1}^{4}\beta_{1}^{4}\eta_{A}^{4}\} \end{array}$$

(25) $$\begin{array}{cc} F_{P}= & \frac{1}{32}\{{\left(1-\eta_{P}\right)}^{4}{\left(\alpha_{1}^{2}\beta_{1}^{2}+\alpha_{1}^{2}\beta_{2}^{2}+\alpha_{2}^{2}\beta_{1}^{2}+\alpha_{2}^{2}\beta_{2}^{2}\right)}^{2}\\ \; & +\eta_{P}^{2}\left(2-\eta_{P}^{2}\right)\alpha_{1}^{4}\beta_{1}^{4}+\eta_{P}^{2}\left(2-\eta_{P}^{2}\right)\alpha_{2}^{4}\beta_{2}^{4}\} \end{array}$$

**Figure 5:**
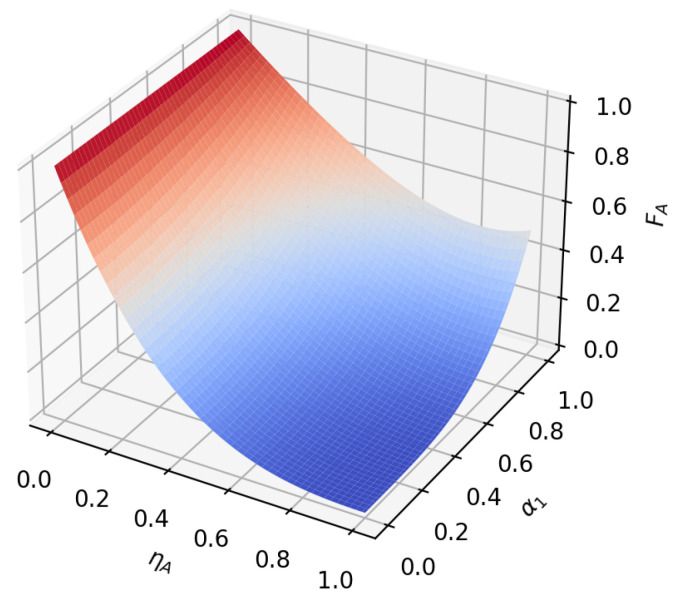
When $\beta_{1}=\frac{\sqrt{2}}{2},\;\beta_{2}=\frac{\sqrt{2}}{2}$, the fidelity under amplitude-damping noise environment variation graph with noise rate and $\alpha_{1}$.

**Figure 6:**
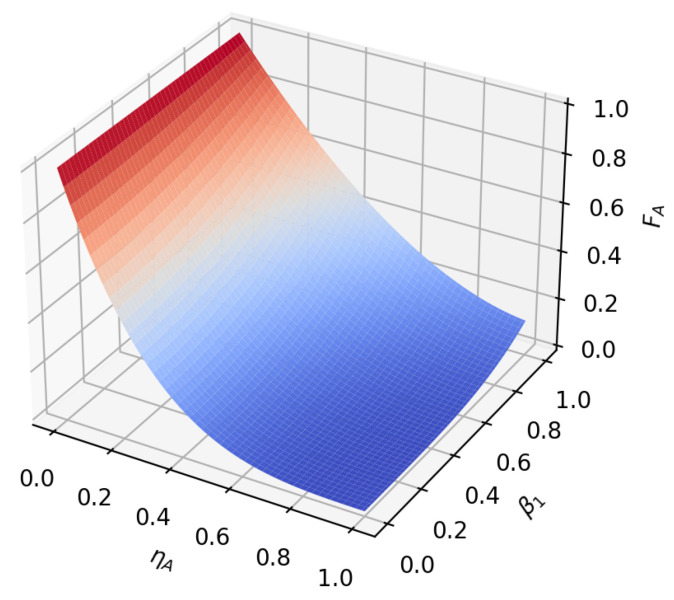
When $\alpha_{1}=\frac{1}{2},\;\alpha_{2}=\frac{\sqrt{3}}{2}$, the fidelity under amplitude-damping noise environment variation graph with noise rate and $\beta_{1}$.

**Figure 7:**
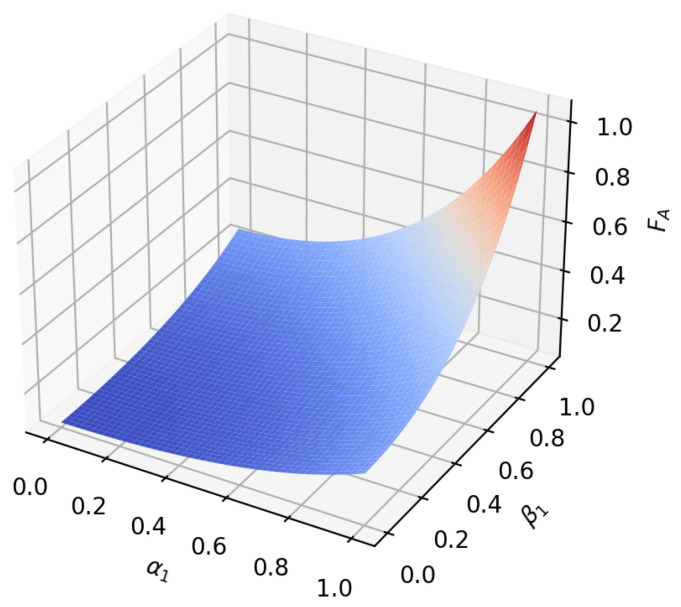
When the noise rate is 0.5, the fidelity changes with $\alpha_{1}$ and $\beta_{1}$ in the amplitude-damping noise environment.

**Figure 8:**
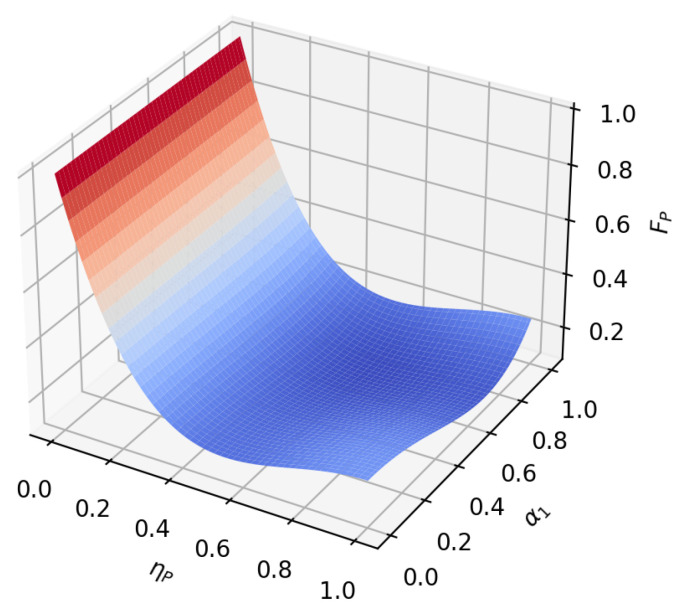
When $\beta_{1}=\frac{\sqrt{2}}{2},\;\beta_{2}=\frac{\sqrt{2}}{2}$, the fidelity under phase-damping noise environment variation graph with noise rate and $\alpha_{1}$.

**Figure 9:**
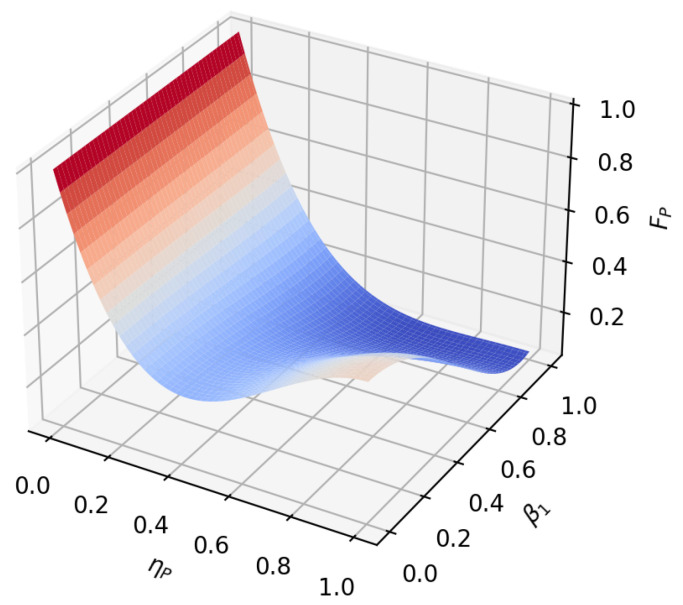
When $\alpha_{1}=\frac{1}{2},\;\alpha_{2}=\frac{\sqrt{3}}{2}$, the fidelity under phase-damping noise environment variation graph with noise rate and $\beta_{1}$.

**Figure 10:**
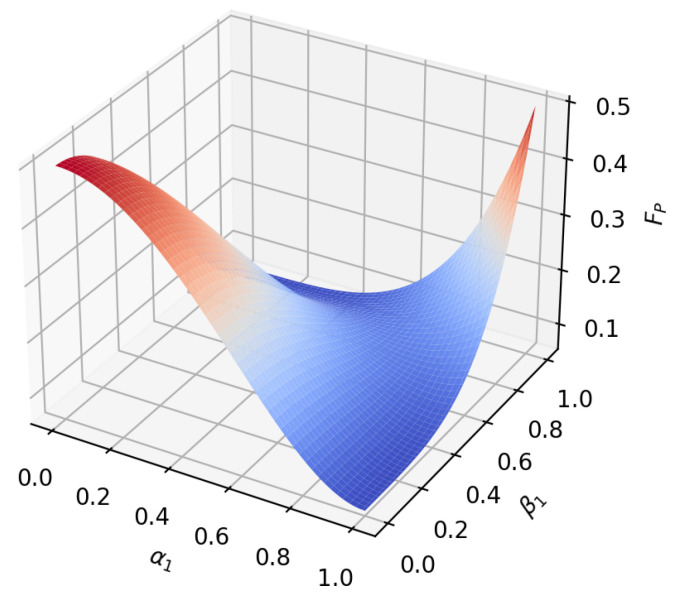
When the noise rate is 0.5, the fidelity changes with $\alpha_{1}$ and $\beta_{1}$ in the phase-damping noise environment.

According to the formula, it can be found that under amplitude-damping noise and phase-damping noise, the fidelity of the transmission process only depends on the amplitude parameter and noise rate of the initial state. To reflect this relationship more intuitively, we plot the relationship between the fidelity function value and the variable under the two noise environments, as shown in Figure 5, Figure 6, Figure 7, Figure 8, Figure 9 and Figure 10. It can be seen from the Figure 5, Figure 6, Figure 8 and Figure 9 that as the noise rate $\eta_{A},\eta_{P}$ increases, the fidelity $F_{A},F_{P}$ gradually reduces.

**Figure 11:**
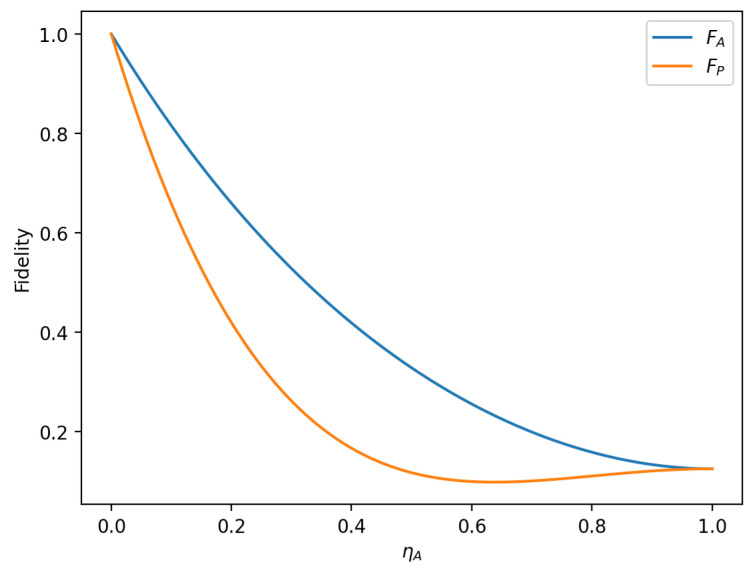
When $\alpha_{1}=\alpha_{2}=\frac{\sqrt{2}}{2},\;\beta_{1}=\beta_{2}=\frac{\sqrt{2}}{2},\;\eta_{A}=\eta_{P}$, fidelity variation with noise rate in amplitude-damping noise environment and phase-damping noise environment.

**Figure 12:**
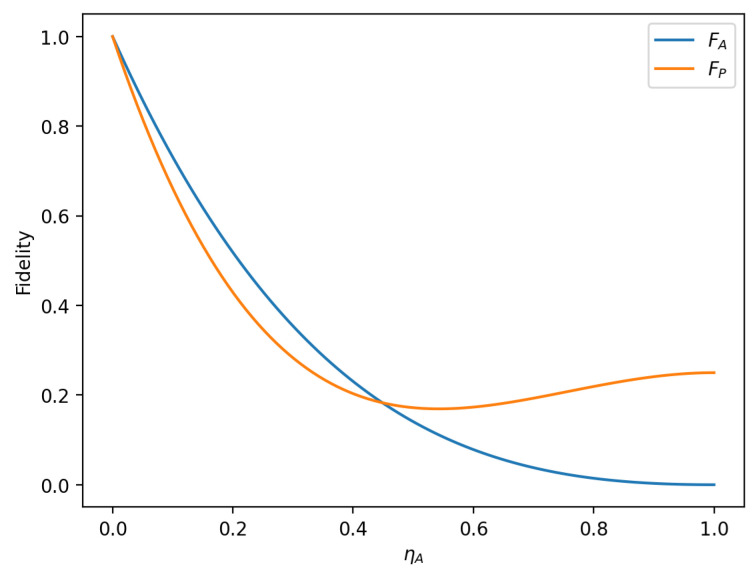
When $\alpha_{1}=\alpha_{2}=\frac{\sqrt{2}}{2},\;\beta_{1}=0,\;\beta_{2}=1,\;\eta_{A}=\eta_{P}$, fidelity variation with noise rate in amplitude-damping noise environment and phase-damping noise environment.

To analyze the influence of the amplitude parameter of the initial state on the fidelity of the hybrid quantum communication protocol, we first set $\alpha_{1}=\alpha_{2}=\frac{\sqrt{2}}{2},\;$$\beta_{1}=\beta_{2}=\frac{\sqrt{2}}{2},\;$$\eta_{A}=\eta_{P}$; the function image of fidelity $F_{A},\;F_{P}$ changing with the noise rate $\eta_{r}$ in two noise environments is represented in Figure 11. Then, setting $\alpha_{1}=\alpha_{2}=\frac{\sqrt{2}}{2},\;\beta_{1}=0,\;\beta_{2}=1,$$\eta_{A}=\eta_{P}$, the image of the fidelity $F_{A},\;F_{P}$ as a function of the noise rate $\eta_{r}$ is plotted for the two noise environments, as shown in Figure 12.

In the case shown in Figure 11, for the same noise rate, the fidelity under the amplitude-damping noise environment (the blue line in Figure 11) is always greater than that under the phase-damping noise environment (the yellow line in Figure 11). In the case shown in Figure 12, when the noise rate $\eta_{A}$ is less than 0.4486, the fidelity under the amplitude-damping noise environment is higher than that under the phase-damping noise environment; when the noise rate $\eta_{A}$ is more significant than 0.4486, the fidelity under the phase-damping noise environment is higher than that under the amplitude-damping noise environment. It can be seen that when the amplitude parameters of the initial state are different, even if the noise rate is the same, there is a significant difference in fidelity for the different noise environments.

This section analyzes the impact of amplitude-damping and phase-damping noise on the fidelity of the hierarchical controlled hybrid quantum communication protocol (HCHQC). The results show that, under the same noise intensity, amplitude-damping noise causes more damage to the fidelity than phase-damping noise, and the parameters of the initial state also affect the trend of the fidelity change. In order to improve the reliability and security of the HCHQC protocol in IoT applications, it is possible to use error correction codes or noise estimation circuits to detect and correct noise errors in the quantum channel or to use noise filters to reduce noise effects.

## 5. Conclusions

This paper proposes a hybrid quantum communication scheme based on six-qubit entangled states for hierarchical control, suitable for specific IoT application scenarios. The scheme realizes the hierarchical control of two communication protocols, quantum teleportation and remote quantum state preparation, on a quantum channel, improving the flexibility and efficiency of communication. This paper describes the principle and steps of the scheme in detail, designs the quantum circuit of the scheme, and performs simulation experiments using IBM’s Qiskit Aer quantum computing simulator. The results show that the fidelity of the scheme reaches 0.999, verifying the feasibility of the scheme. This paper also analyzes the factors that affect the fidelity of the scheme in a specific quantum noise environment, providing references for further optimization of the scheme. By combining quantum communication technology with IoT application scenarios, this paper provides a new possibility for IoT communication. In order to improve the efficiency and reliability of quantum communication, we will continue to conduct in-depth research on high-dimensional entangled states, complex communication protocols, noise models and error correction mechanisms in the future and seek better solutions.

## Figures and Tables

**Table 1:** Alice’s measurement results and the corresponding state of the remaining qubits.

Alice’s Measurement Result	State of the Remaining Qubits
${|\Psi^{1}\rangle }_{a,\;A_{1}}=\frac{1}{\sqrt{2}}\left(|00\rangle +|11\rangle \right)$	$\begin{array}{c} \begin{array}{cc} \langle \Psi^{1}||\tau \rangle & =\frac{1}{2\sqrt{2}}(\alpha_{1}|00000\rangle -\alpha_{1}|01111\rangle \\ & +\alpha_{2}|10010\rangle -\alpha_{2}{|11101\rangle )}_{B_{1}A_{2}B_{2}CD} \end{array} \end{array}$
${|\Psi^{2}\rangle }_{a,A_{1}}=\frac{1}{\sqrt{2}}\left(|00\rangle -|11\rangle \right)$	$\begin{array}{c} \begin{array}{cc} \langle \Psi^{2}||\tau \rangle & =\frac{1}{2\sqrt{2}}(\alpha_{1}|00000\rangle -\alpha_{1}|01111\rangle \\ & -\alpha_{2}|10010\rangle +\alpha_{2}{|11101\rangle )}_{B_{1}A_{2}B_{2}CD} \end{array} \end{array}$
${|\Psi^{3}\rangle }_{a,A_{1}}=\frac{1}{\sqrt{2}}\left(|01\rangle +|10\rangle \right)$	$\begin{array}{c} \begin{array}{cc} \langle \Psi^{3}||\tau \rangle & =\frac{1}{2\sqrt{2}}(\alpha_{1}|10010\rangle -\alpha_{1}|11101\rangle \\ & +\alpha_{2}|00000\rangle -\alpha_{2}{|01111\rangle )}_{B_{1}A_{2}B_{2}CD} \end{array} \end{array}$
${|\Psi^{4}\rangle }_{a,A_{1}}=\frac{1}{\sqrt{2}}\left(|01\rangle -|10\rangle \right)$	$\begin{array}{c} \begin{array}{cc} \langle \Psi^{4}||\tau \rangle & =\frac{1}{2\sqrt{2}}(\alpha_{1}|10010\rangle -\alpha_{1}|11101\rangle \\ & -\alpha_{2}|00000\rangle +\alpha_{2}{|01111\rangle )}_{B_{1}A_{2}B_{2}CD} \end{array} \end{array}$

**Table 2:** The measurement results of Alice and Bob and the state of the remaining qubits.

Alice’s Measurement Result	Bob’s Measurement Result	State of the Remaining Qubits
${|\Psi^{1}\rangle }_{a,A_{1}}=\frac{1}{\sqrt{2}}\left(|00\rangle +|11\rangle \right)$	${|\Phi^{1}\rangle }_{B_{2}}$	$\begin{array}{c} \begin{array}{cc} \langle \Phi^{1}|\langle \Psi^{1}||\tau \rangle & =\frac{1}{2\sqrt{2}}(\alpha_{1}\beta_{1}|0000\rangle -\alpha_{1}\beta_{2}|0111\rangle +\alpha_{2}\beta_{1}|1010\rangle \\ & -\alpha_{2}\beta_{2}{|1101\rangle )}_{B_{1}A_{2}CD} \end{array} \end{array}$
	${|\Phi^{2}\rangle }_{B_{2}}$	$\begin{array}{c} \begin{array}{cc} \langle \Phi^{2}|\langle \Psi^{1}||\tau \rangle & =\frac{1}{2\sqrt{2}}(\alpha_{1}\beta_{2}|0000\rangle +\alpha_{1}\beta_{1}|0111\rangle +\alpha_{2}\beta_{2}|1010\rangle \\ & +\alpha_{2}\beta_{1}{|1101\rangle )}_{B_{1}A_{2}CD} \end{array} \end{array}$
${|\Psi^{2}\rangle }_{a,A_{1}}=\frac{1}{\sqrt{2}}\left(|00\rangle -|11\rangle \right)$	${|\Phi^{1}\rangle }_{B_{2}}$	$\begin{array}{c} \begin{array}{cc} \langle \Phi^{1}|\langle \Psi^{2}||\tau \rangle & =\frac{1}{2\sqrt{2}}(\alpha_{1}\beta_{1}|0000\rangle -\alpha_{1}\beta_{2}|0111\rangle -\alpha_{2}\beta_{1}|1010\rangle \\ & +\alpha_{2}\beta_{2}{|1101\rangle )}_{B_{1}A_{2}CD} \end{array} \end{array}$
	${|\Phi^{2}\rangle }_{B_{2}}$	$\begin{array}{c} \begin{array}{cc} \langle \Phi^{2}|\langle \Psi^{2}||\tau \rangle & =\frac{1}{2\sqrt{2}}(\alpha_{1}\beta_{2}|0000\rangle +\alpha_{1}\beta_{1}|0111\rangle -\alpha_{2}\beta_{2}|1010\rangle \\ & -\alpha_{2}\beta_{1}{|1101\rangle )}_{B_{1}A_{2}CD} \end{array} \end{array}$
${|\Psi^{3}\rangle }_{a,A_{1}}=\frac{1}{\sqrt{2}}\left(|01\rangle +|10\rangle \right)$	${|\Phi^{1}\rangle }_{B_{2}}$	$\begin{array}{c} \begin{array}{cc} \langle \Phi^{1}|\langle \Psi^{3}||\tau \rangle & =\frac{1}{2\sqrt{2}}(\alpha_{1}\beta_{1}|1010\rangle -\alpha_{1}\beta_{2}|1101\rangle +\alpha_{2}\beta_{1}|0000\rangle \\ & -\alpha_{2}\beta_{2}{|0111\rangle )}_{B_{1}A_{2}CD} \end{array} \end{array}$
	${|\Phi^{2}\rangle }_{B_{2}}$	$\begin{array}{c} \begin{array}{cc} \langle \Phi^{2}|\langle \Psi^{3}||\tau \rangle & =\frac{1}{2\sqrt{2}}(\alpha_{1}\beta_{2}|1010\rangle +\alpha_{1}\beta_{1}|1101\rangle +\alpha_{2}\beta_{2}|0000\rangle \\ & +\alpha_{2}\beta_{1}{|0111\rangle )}_{B_{1}A_{2}CD} \end{array} \end{array}$
${|\Psi^{4}\rangle }_{a,A_{1}}=\frac{1}{\sqrt{2}}\left(|01\rangle -|10\rangle \right)$	${|\Phi^{1}\rangle }_{B_{2}}$	$\begin{array}{c} \begin{array}{cc} \langle \Phi^{1}|\langle \Psi^{4}||\tau \rangle & =\frac{1}{2\sqrt{2}}(\alpha_{1}\beta_{1}|1010\rangle -\alpha_{1}\beta_{2}|1101\rangle -\alpha_{2}\beta_{1}|0000\rangle \\ & +\alpha_{2}\beta_{2}{|0111\rangle )}_{B_{1}A_{2}CD} \end{array} \end{array}$
	${|\Phi^{2}\rangle }_{B_{2}}$	$\begin{array}{c} \begin{array}{cc} \langle \Phi^{2}|\langle \Psi^{4}||\tau \rangle & =\frac{1}{2\sqrt{2}}(\alpha_{1}\beta_{2}|1010\rangle +\alpha_{1}\beta_{1}|1101\rangle -\alpha_{2}\beta_{2}|0000\rangle \\ & -\alpha_{2}\beta_{1}{|0111\rangle )}_{B_{1}A_{2}CD} \end{array} \end{array}$

**Table 3:** Charlie’s measurement results and Bob’s unitary operation corresponding to each result.

State before Charlie Measured	Charlie’s Measurement Result	State of the Remaining Qubits	Bob’s Operation
$\langle \Phi^{1}|\langle \Psi^{1}||\tau \rangle$	$|\nu^{1}\rangle$	$\left(\alpha_{1}|0\rangle +\alpha_{2}|1\rangle \right)B_{1}⊗\left(\beta_{1}|00\rangle -\beta_{2}|11\rangle \right)A_{2}D$	*I*
	$|\nu^{2}\rangle$	$\left(\alpha_{1}|0\rangle -\alpha_{2}|1\rangle \right)B_{1}⊗\left(\beta_{1}|00\rangle +\beta_{2}|11\rangle \right)A_{2}D$	$\sigma_{z}$
$\langle \Phi^{2}|\langle \Psi^{1}||\tau \rangle$	$|\nu^{1}\rangle$	$\left(\alpha_{1}|0\rangle +\alpha_{2}|1\rangle \right)B_{1}⊗\left(\beta_{2}|00\rangle +\beta_{1}|11\rangle \right)A_{2}D$	*I*
	$|\nu^{2}\rangle$	$\left(\alpha_{1}|0\rangle -\alpha_{2}|1\rangle \right)B_{1}⊗\left(\beta_{2}|00\rangle -\beta_{1}|11\rangle \right)A_{2}D$	$\sigma_{z}$
$\langle \Phi^{1}|\langle \Psi^{2}||\tau \rangle$	$|\nu^{1}\rangle$	$\left(\alpha_{1}|0\rangle -\alpha_{2}|1\rangle \right)B_{1}⊗\left(\beta_{1}|00\rangle -\beta_{2}|11\rangle \right)A_{2}D$	$\sigma_{z}$
	$|\nu^{2}\rangle$	$\left(\alpha_{1}|0\rangle +\alpha_{2}|1\rangle \right)B_{1}⊗\left(\beta_{1}|00\rangle +\beta_{2}|11\rangle \right)A_{2}D$	*I*
$\langle \Phi^{2}|\langle \Psi^{2}||\tau \rangle$	$|\nu^{1}\rangle$	$\left(\alpha_{1}|0\rangle -\alpha_{2}|1\rangle \right)B_{1}⊗\left(\beta_{2}|00\rangle +\beta_{1}|11\rangle \right)A_{2}D$	$\sigma_{z}$
	$|\nu^{2}\rangle$	$\left(\alpha_{1}|0\rangle +\alpha_{2}|1\rangle \right)B_{1}⊗\left(\beta_{2}|00\rangle -\beta_{1}|11\rangle \right)A_{2}D$	*I*
$\langle \Phi^{1}|\langle \Psi^{3}||\tau \rangle$	$|\nu^{1}\rangle$	$\left(\alpha_{2}|0\rangle +\alpha_{1}|1\rangle \right)B_{1}⊗\left(\beta_{1}|00\rangle -\beta_{2}|11\rangle \right)A_{2}D$	$\sigma_{x}$
	$|\nu^{2}\rangle$	$\left(\alpha_{2}|0\rangle -\alpha_{1}|1\rangle \right)B_{1}⊗\left(\beta_{1}|00\rangle +\beta_{2}|11\rangle \right)A_{2}D$	$-i\sigma_{y}$
$\langle \Phi^{2}|\langle \Psi^{3}||\tau \rangle$	$|\nu^{1}\rangle$	$\left(\alpha_{2}|0\rangle +\alpha_{1}|1\rangle \right)B_{1}⊗\left(\beta_{2}|00\rangle +\beta_{1}|11\rangle \right)A_{2}D$	$\sigma_{x}$
	$|\nu^{2}\rangle$	$\left(\alpha_{2}|0\rangle -\alpha_{1}|1\rangle \right)B_{1}⊗\left(\beta_{2}|00\rangle -\beta_{1}|11\rangle \right)A_{2}D$	$-i\sigma_{y}$
$\langle \Phi^{1}|\langle \Psi^{4}||\tau \rangle$	$|\nu^{1}\rangle$	$\left(-\alpha_{2}|0\rangle +\alpha_{1}|1\rangle \right)B_{1}⊗\left(\beta_{1}|00\rangle -\beta_{2}|11\rangle \right)A_{2}D$	$i\sigma_{y}$
	$|\nu^{2}\rangle$	$\left(-\alpha_{2}|0\rangle -\alpha_{1}|1\rangle \right)B_{1}⊗\left(\beta_{1}|00\rangle +\beta_{2}|11\rangle \right)A_{2}D$	$-\sigma_{x}$
$\langle \Phi^{2}|\langle \Psi^{4}||\tau \rangle$	$|\nu^{1}\rangle$	$\left(-\alpha_{2}|0\rangle +\alpha_{1}|1\rangle \right)B_{1}⊗\left(\beta_{2}|00\rangle +\beta_{1}|11\rangle \right)A_{2}D$	$i\sigma_{y}$
	$|\nu^{2}\rangle$	$\left(-\alpha_{2}|0\rangle -\alpha_{1}|1\rangle \right)B_{1}⊗\left(\beta_{2}|00\rangle -\beta_{1}|11\rangle \right)A_{2}D$	$-\sigma_{x}$

**Table 4:** David’s measurement results and Alice’s unitary operation corresponding to each result.

State before David Measured	David’s Measurement Result	State of Qubits	Alice’s Operation
$\langle \nu^{1}|\langle \Phi^{1}|\langle \Psi^{1}||\tau \rangle$	$|\nu^{1}\rangle$	$\beta_{1}|0\rangle -\beta_{2}|1\rangle$	$\sigma_{z}$
	$|\nu^{2}\rangle$	$\beta_{1}|0\rangle +\beta_{2}|1\rangle$	*I*
$\langle \nu^{2}|\langle \Phi^{1}|\langle \Psi^{1}||\tau \rangle$	$|\nu^{1}\rangle$	$\beta_{1}|0\rangle +\beta_{2}|1\rangle$	*I*
	$|\nu^{2}\rangle$	$\beta_{1}|0\rangle -\beta_{2}|1\rangle$	$\sigma_{z}$
$\langle \nu^{1}|\langle \Phi^{2}|\langle \Psi^{1}||\tau \rangle$	$|\nu^{1}\rangle$	$\beta_{2}|0\rangle +\beta_{1}|1\rangle$	$\sigma_{x}$
	$|\nu^{2}\rangle$	$\beta_{2}|0\rangle -\beta_{1}|1\rangle$	$-i\sigma_{y}$
$\langle \nu^{2}|\langle \Phi^{2}|\langle \Psi^{1}||\tau \rangle$	$|\nu^{1}\rangle$	$\beta_{2}|0\rangle -\beta_{1}|1\rangle$	$-i\sigma_{y}$
	$|\nu^{2}\rangle$	$\beta_{2}|0\rangle +\beta_{1}|1\rangle$	$\sigma_{x}$
$\langle \nu^{1}|\langle \Phi^{1}|\langle \Psi^{2}||\tau \rangle$	$|\nu^{1}\rangle$	$\beta_{1}|0\rangle -\beta_{2}|1\rangle$	$\sigma_{z}$
	$|\nu^{2}\rangle$	$\beta_{1}|0\rangle +\beta_{2}|1\rangle$	*I*
$\langle \nu^{2}|\langle \Phi^{1}|\langle \Psi^{2}||\tau \rangle$	$|\nu^{1}\rangle$	$\beta_{1}|0\rangle +\beta_{2}|1\rangle$	*I*
	$|\nu^{2}\rangle$	$\beta_{1}|0\rangle -\beta_{2}|1\rangle$	$\sigma_{z}$
$\langle \nu^{1}|\langle \Phi^{2}|\langle \Psi^{2}||\tau \rangle$	$|\nu^{1}\rangle$	$\beta_{2}|0\rangle +\beta_{1}|1\rangle$	$\sigma_{x}$
	$|\nu^{2}\rangle$	$\beta_{2}|0\rangle -\beta_{1}|1\rangle$	$-i\sigma_{y}$
$\langle \nu^{2}|\langle \Phi^{2}|\langle \Psi^{2}||\tau \rangle$	$|\nu^{1}\rangle$	$\beta_{2}|0\rangle -\beta_{1}|1\rangle$	$-i\sigma_{y}$
	$|\nu^{2}\rangle$	$\beta_{2}|0\rangle +\beta_{1}|1\rangle$	$\sigma_{x}$
$\langle \nu^{1}|\langle \Phi^{1}|\langle \Psi^{3}||\tau \rangle$	$|\nu^{1}\rangle$	$\beta_{1}|0\rangle -\beta_{2}|1\rangle$	$\sigma_{z}$
	$|\nu^{2}\rangle$	$\beta_{1}|0\rangle +\beta_{2}|1\rangle$	*I*
$\langle \nu^{2}|\langle \Phi^{1}|\langle \Psi^{3}||\tau \rangle$	$|\nu^{1}\rangle$	$\beta_{1}|0\rangle +\beta_{2}|1\rangle$	*I*
	$|\nu^{2}\rangle$	$\beta_{1}|0\rangle -\beta_{2}|1\rangle$	$\sigma_{z}$
$\langle \nu^{1}|\langle \Phi^{2}|\langle \Psi^{3}||\tau \rangle$	$|\nu^{1}\rangle$	$\beta_{2}|0\rangle +\beta_{1}|1\rangle$	$\sigma_{x}$
	$|\nu^{2}\rangle$	$\beta_{2}|0\rangle -\beta_{1}|1\rangle$	$-i\sigma_{y}$
$\langle \nu^{2}|\langle \Phi^{2}|\langle \Psi^{3}||\tau \rangle$	$|\nu^{1}\rangle$	$\beta_{2}|0\rangle -\beta_{1}|1\rangle$	$-i\sigma_{y}$
	$|\nu^{2}\rangle$	$\beta_{2}|0\rangle +\beta_{1}|1\rangle$	$\sigma_{x}$
$\langle \nu^{1}|\langle \Phi^{1}|\langle \Psi^{4}||\tau \rangle$	$|\nu^{1}\rangle$	$\beta_{1}|0\rangle -\beta_{2}|1\rangle$	$\sigma_{z}$
	$|\nu^{2}\rangle$	$\beta_{1}|0\rangle +\beta_{2}|1\rangle$	*I*
$\langle \nu^{2}|\langle \Phi^{1}|\langle \Psi^{4}||\tau \rangle$	$|\nu^{1}\rangle$	$\beta_{1}|0\rangle +\beta_{2}|1\rangle$	*I*
	$|\nu^{2}\rangle$	$\beta_{1}|0\rangle -\beta_{2}|1\rangle$	$\sigma_{z}$
$\langle \nu^{1}|\langle \Phi^{2}|\langle \Psi^{4}||\tau \rangle$	$|\nu^{1}\rangle$	$\beta_{2}|0\rangle +\beta_{1}|1\rangle$	$\sigma_{x}$
	$|\nu^{2}\rangle$	$\beta_{2}|0\rangle -\beta_{1}|1\rangle$	$-i\sigma_{y}$
$\langle \nu^{2}|\langle \Phi^{2}|\langle \Psi^{4}||\tau \rangle$	$|\nu^{1}\rangle$	$\beta_{2}|0\rangle -\beta_{1}|1\rangle$	$-i\sigma_{y}$
	$|\nu^{2}\rangle$	$\beta_{2}|0\rangle +\beta_{1}|1\rangle$	$\sigma_{x}$

**Table 5:** Alice, Bob, David, and Charlie’s measurement results and corresponding classical information.

Measurer	Measurement Result	Classical Information
Alice	${|\Psi^{1}\rangle }_{a,A_{1}}=\frac{1}{\sqrt{2}}\left(|00\rangle +|11\rangle \right)$	00
	${|\Psi^{2}\rangle }_{a,A_{1}}=\frac{1}{\sqrt{2}}\left(|00\rangle -|11\rangle \right)$	01
	${|\Psi^{3}\rangle }_{a,A_{1}}=\frac{1}{\sqrt{2}}\left(|01\rangle +|10\rangle \right)$	10
	${|\Psi^{4}\rangle }_{a,A_{1}}=\frac{1}{\sqrt{2}}\left(|01\rangle -|10\rangle \right)$	11
Bob	${|\Phi^{1}\rangle }_{B_{2}}=\beta_{1}|0\rangle +\beta_{2}|1\rangle$	0
	${|\Phi^{2}\rangle }_{B_{2}}=\beta_{2}|0\rangle -\beta_{1}|1\rangle$	1
Charlie/David	$|\nu^{1}\rangle =\frac{1}{\sqrt{2}}\left(|0\rangle +|1\rangle \right)$	0
	$|\nu^{2}\rangle =\frac{1}{\sqrt{2}}\left(|0\rangle -|1\rangle \right)$	1

**Table 6:** Transmission result.

Protocal	States	Shots	Frequency (%)
QTP	|0〉	4098	50.02%
	|1〉	4094	49.97%
RSP	|0〉	2696	32.91%
	|1〉	5496	67.08%

## Data Availability

Data are contained within the article.
